# Psychometric properties of Leisure Satisfaction Scale (LSS)-short form: a Rasch rating model calibration approach

**DOI:** 10.1186/s40359-022-00861-1

**Published:** 2022-06-15

**Authors:** Sae-Hyung Kim, Dongwook Cho

**Affiliations:** 1 Department of Physical Education, Chungbuk National University, Chungdae-rol 1, Seowon-gu, Chungbuk 28644 South Korea; 2 Department of Health, Physical Education and Recreation, Alcorn State University, 1000 ASU Drive #1380, Lorman, MS 39096 USA

**Keywords:** Differential item functioning, Evaluation, Instrument development, Item fit, Leisure satisfaction, Person-item map, Rasch measurement, Validity

## Abstract

**Background:**

Leisure satisfaction has been one of primary variables to explain an individual’s choice of leisure and recreational activities’ participation. The Leisure Satisfaction Scale (LSS)-short form has been widely utilized to measure leisure and recreation participants’ satisfaction levels. However, limited research has been studied on the LSS-short form that would provide sufficient evidence to use it to measure individual leisure satisfaction levels. Thus, the purpose of the study was to determine whether the LSS-short form would be appropriate to measure individuals’ leisure satisfaction levels.

**Method:**

The convenience sampling was used in this study from the south-central United States. The LSS-short form questionnaire was administered to 436 individuals after removing 20 surveys due to incomplete questions. The WINSTEPS computer program was utilized to analyze the Rating scale fit; Item fit; Differential Item Functioning (DIF); and Person-Item map by utilizing Rasch rating scale model.

**Results:**

The results indicated that the five-point Likert-type LSS-short form was appropriate to utilize. Two of 24 LSS-short form items had overfit or misfit and were eliminated. DIF indicated that all remained 22 items were suitable to measure leisure satisfaction levels. Overall, 22 item were finally selected for the reconstructed version of the LSS-short form. In addition, Person-Item map showed that ability and item difficulty were fit matched.

**Conclusions:**

As the importance of leisure has been increased, the newly reconstructed LSS-short form would be recommended to evaluate individual leisure satisfaction levels in future studies. Furthermore, leisure and recreation professionals can provide and develop effective leisure activities or programs by measuring individual’s leisure satisfaction level with the new version of LSS-short form.

## Introduction

The In modern society, leisure activities are sometimes considered more important than work, as more individuals enjoy leisure activities such as exercise, recreational activities, and sport participation through the remarkable increase in leisure time. According to the US Department of Labor [1], 95.6% of respondents spent even a short amount of time per day for leisure activities, utilizing an average of 5.27 h per day for leisure activities. The most common leisure activity was watching television (2.77 h/day), followed by social activities (0.65 h/day) and exercise and sport participation (0.29 h/day). Demographically, older adults aged 65 and over enjoyed leisure activities most frequently (7.41 h/day), while those aged 35–44 years old provided leisure activities only 4.08 h per day. In addition, men (5.69 h/day) spent about 49 min more on leisure activities than women (4.87 h/day) [1].

Leisure can be described as an experience which is internally motivated and free from work or other mandatory activities [2, 3]. Cordes and Ibrahim [4] described the three essential elements of leisure as perceived freedom on the experience at will, autotelic activity with intrinsic motivation, and beneficial outcome through leisure activities. As such, leisure can be comprehensively determined by active activities such as sports participations and tourism, as well as passive activities including reading and meditation [5, 6].

As the number of leisure activity participants has increased, many studies that examine how leisure activities affect participants’ satisfaction have been conducted. One study determined that participation in exercise and physical activities during leisure time was a statistically significant predictor of older adults’ life satisfaction levels [5]. The study by Ayyildi and Gokyurek [7] indicated that participation in recreational dance activities and leisure satisfaction were statistically related based on age group, income and education level, marital status, and parental status. Another study showed the significant relationship between family leisure satisfaction and satisfaction with family life [8]. Thus, results from these studies have indicated that participation in leisure activities were closely connected to physical, psychological, and social benefits and satisfaction [3, 9, 10].

To support this research, instruments and questionnaires have been created and developed to measure participants’ satisfaction levels [11, 12]. Among these methods, Beard and Ragheb [11] created and developed the Leisure Satisfaction Scale (LSS) to measure individual leisure satisfaction levels. The LSS is composed of 51 items and each item is measured utilizing a five-point Likert-type scale from 1 (strongly disagree) to 5 (strongly agree) in which the higher scores are higher leisure satisfaction levels. More specifically, these 51 LSS items form up to six subscales including: psychological (13 items), educational (12 items), social (11 items), relaxation (4 items), physiological (6 items), and aesthetic (5 items) satisfaction. At this time, reliability test of LSS was assessed by administrating the sample of 603 individuals, which included students, professionals, technical workers, and retirees. Then, it was assessed with another sample of 347 subjects, after which some changes and refinements were administrated. Through the assessment of two samples, this instrument demonstrated it was reliable for measuring individual leisure satisfaction level. Further, Beard and Ragheb [11] sampled four items from each subscale to reduce the approximate 20 min measurement time of the LSS. They developed 24 items into the LSS-short form, which reduced measurement time to no more than 10 min and attained an internal consistency of 0.93.

Several studies have utilized the LSS and LSS-short form to conduct research into leisure satisfaction [13–17]. However, there have only been limited studies to indicate the validity of the LSS and LSS-short form. Originally, validity of the LSS was evaluated by approximately 160 leisure and recreation professionals and was generally suggested to be useful tool. However, their responses can be interpreted as the logical validity method in which the scale emerges as a metric to measure leisure satisfaction [11]. Recently, Trotter et al. [18] conducted research that compared the Adolescent Leisure Interest Profile (ALIP) developed by Henry [12] with the LSS-short form by using a group of 37 adolescent subjects. The results indicated that concurrent validity between LSS-short form and ALIP was found to be very weak or unrelated. Another study measured the validity of LSS-short form by estimating from 515 Korean college students and adults that the results showed the LSS would be appropriate to utilize [19]. However, there has been limited research on the LSS-short form that would provide sufficient evidence to utilize it to measure individual leisure satisfaction levels.

The Rasch rating scale model based on Item Response Theory (IRT) has been suggested because the evidence showed that the Rasch rating scale model can reduce more errors by removing statistically irrelevant items [20, 21]. Therefore, the purpose of this study aimed to reevaluate the LSS-short form questionnaires so that it would be appropriate to apply them to measure individual leisure satisfaction levels by utilizing the Rasch rating scale model: (1) Rating scale fit; (2) Item fit; (3) Differential Item Functioning (DIF); and (4) Person-Item map.

## Methods

### Participants

The non-probability convenience sampling was utilized that participants in this study were recruited from the south-central United States. The respondents were informed of the recognition of anonymity agreement and voluntary participation by signing the Informed consent form for the survey, and that prior to testing proper approval had been obtained from the Institutional Review Board (IRB) of the higher education institution of the lead researcher. A total of 456 participants completed the survey, but due to incomplete questions on 20 surveys, a total of 436 questionnaires were utilized for the Rasch rating scale model analysis. The participants were composed of 236 women and 200 men between the ages of 18 and 76 years.

### The Leisure satisfaction sale (LSS)-short form

The LSS-short form questionnaire was utilized in this research [11]. The LSS-short form was composed of 24 items with a five-point Likert-type scale (1: strongly disagree–5: strongly agree). These items were further comprised of six subscales, which are scored by calculating the mean of four items of a subscale. These subscales include psychological (item No. 1–4), educational (item No. 5–8), social (item No. 9–12), relaxation (item No. 13–16), physiological (item No. 17–20), and aesthetic (item No. 21–24), as shown in Table 1.

**Table 1:** Leisure Satisfaction Scale (LSS)-short form

Subscale	Item
Psychological	1. My leisure activities are very interesting to me
	2. My leisure activities give me self-confidence
	3. My leisure activities give me a sense of accomplishment
	4. I use many different skills and abilities in my leisure activities
Educational	5. My leisure activities increase my knowledge about things around me
	6. My leisure activities provide opportunities to try new things
	7. My leisure activities help me to learn about myself
	8. My leisure activities help me to learn about other people
Social	9. I have social interaction with others through leisure activities
	10. My leisure activities have helped me to develop close relationship with others
	11. The people I meet in my leisure activities are friendly
	12. I associated with people in my free time who enjoy doing leisure activities a great deal
Relaxation	13. My leisure activities help me to relax
	14. My leisure activities help relieve stress
	15. My leisure activities contribute to my emotional well being
	16. I engage in leisure activities simply because I like doing them
Physiological	17. My leisure activities are physically challenging
	18. I do leisure activities which develop my physical fitness
	19. I do leisure activities which restore me physically
	20. My leisure activities help me to stay healthy
Aesthetic	21. The areas or places where I engage in my leisure activities are fresh and clean
	22. The areas or places where I engage in my leisure activities are interesting
	23. The areas or places where I engage in my leisure activities are beautiful
	24. The areas or places where I engage in my leisure activities are well designed

### Data analysis

The Rasch model was applied to reevaluate the LSS-short form followed by content validity was confirmed by experts of leisure, recreation, and psychology. In particular, the Rasch rating scale model of the Rasch model by Andrich [22] was utilized. However, prior to reevaluating the LSS-short form, it was necessary to confirm whether 24 items violated the basic unidimensional assumption of the Rasch rating scale model test. It was suggested that items would be properly examined for unidimensionality if more than 20% of the total variance was determined by the eigenvalue variance of the first component accounts by applying the Principal Component Analysis (PCA) [23]. Additionally, another assumption of Rasch model, local independence was automatically examined when unidimensionality was satisfied with these items [24]. Reliability of 24 items of the LSS-short form was confirmed by Cronbach alpha coefficient at 0.901.

Thus, the unidimensionality of the LSS-short form was analyzed by utilizing the Statistical Package for the Social Sciences 24 (SPSS). The results revealed an eigenvalue variance of 44.04%, meaning that the 24 items of the LSS-short form were satisfactory for the basic assumption of unidimensionality. Furthermore, the WINSTEPS 4.4.4 computer program was utilized to analyze Rating scale fit, Item fit, and Differential Item Functioning (DIF) of the Rasch rating scale model.

#### Rating scale fit

The Rating scale fit of Rasch rating scale model was applied to assess the 24 items’ suitability for a five-point Likert-type LSS-short form by utilizing the category probability curve and the derivation index. The criteria of rating scale fit were to determine whether: each category label was counted more than 10 times in total; whether the average estimated value of each category was sequenced; whether the outfit of each category was less than 2.0; and whether the step calibration increased in the order of determined category, which represents the intersection of the category characteristic curves. Thus, it was examined whether a five-point Likert-type LSS-short form would be suitable to utilize when all four of the above criteria are validated [22].

#### Item fit

The Item fit test of the Rasch rating scale model was examined to estimate misfit or overfit items that determined the items which were too confused or too easy to respond. The results of this test indicated how consistent the degree of difficulty is for each item and by following the $x^{z}$ distribution with the expected value of 1.00. If the fit index logit of each item is closer to the expected value (1.00), the analytical data is appropriate to utilize for the model. It would be regarded as a bias item when an expected value of an item is more than 1.50 (misfit). More specifically, the local independence and unidimensionality were examined when an expected value of each item’s infit and outfit is between 0.50 and 1.50 [25].

#### Differential item functioning (DIF)

Differential Item Functioning (DIF) of the Rasch rating scale model was utilized to view whether the property of individual items was appropriated or not. It is valuable to utilize DIF by extracting an unexpected behavior of items in order to approve the generalized validity through separating by subgroups such as age, education, or sex [26, 27]. In this study, the systematically biased items were examined based on the characteristics of male and female groups by analyzing the Rasch-Welch (RW) t-test, p-value probability, DIF contrast value and category status when person’s ability for each item was fixed as “0”. Specifically, this study utilized the DIF contrast value and category status which was examined in terms of probability based on the five DIF levels [28]. This DIF category status indicated it should be regarded as negligible bias (Class A) if the absolute value is 0.00–0.42. If the value is between 0.43 and 0.63, it suggested a slight to moderate bias (Class B). Finally, it suggested a moderate to large bias (Class C) if the value is more than 0.64. It is also suggested DIF items in Class B or C are set as ‘+’ if those favor the focal group, while it is set as ‘−‘if items favor the reference group [28]. In this study, the male participant group was set as the focal group and the female group as the reference group.

#### Person-item map

The final evaluation step was the Person-Item map of the Rasch rating scale model, which only was able to verify whether the reconstructed LSS-short form was appropriate to utilize. It was applied to estimate the item difficulty of the reconstructed LSS-short form through a comparison between the person ability and item difficulty. These two were directly compared by covering these on the same logit scale. If the two distributions of a person’s ability and item difficulty are closer, it will be a more suitable questionnaire to utilize [29]. Furthermore, the Person's Separation Reliability index (PSR index) was applied to analyze Person-Item map with the numerical index range of 0–1. The better distribution was examined if the reconstructed LSS-short form was closer to 1 [24].

## Results

### Rasting scale fit

The suitability of the five-point Likert-type LSS-short form was verified by utilizing the four criteria for each of the five categories: an observed count, an average expectation by size, an outfit, and a step calibration. The results revealed that the observed count for each category was selected at least 10 times or more, and the averaged expectation was sequenced based on the category level. The outfit values for each category showed less than 2.0 and the step calibration values increased in the order of the determined category level from 1 to 5. Furthermore, it was assumed that a five-point Likert-type LSS-short form was appropriate to utilize (Table 2).

**Table 2:** Suitability of five-point likert-type LSS-short form

Category label	Observed count	Average expect	Outfit	Step calibration
1	352	− 1.57	1.79	None
2	849	− 0.32	1.03	− 1.84
3	2894	0.52	1.01	− 1.11
4	4160	1.26	0.84	0.53
5	2209	2.26	0.97	2.43

### Item fit

Twenty-four items of the LSS-short form was analyzed by calculating item fit scores through the Rasch rating scale model. The results revealed that an expected value of item 17, “My leisure activities are physically challenging” (infit = 1.91; outfit = 2.28) and item 18, “I do leisure activities which develop my physical fitness” (infit = 1.47; outfit = 1.57), part of the subscale of “Physiological” benefits, showed more than a 1.50 (misfit) (Table 3).

**Table 3:** Item fit of LSS-short form

Items No	Logit	Infit	Outfit
1	− 0.59	0.97	1.07
2	0.70	0.80	0.94
3	0.33	0.74	0.75
4	0.36	0.91	0.93
5	− 0.51	0.97	0.95
6	− 0.93	0.91	0.88
7	0.51	0.86	0.88
8	− 0.36	0.92	0.93
9	− 0.60	0.81	0.80
10	− 0.25	0.96	0.95
11	− 0.46	0.84	0.87
12	− 0.19	0.96	0.95
13	− 0.38	1.04	1.14
14	− 0.38	1.11	1.19
15	− 0.04	1.05	1.07
16	− 0.69	0.99	0.97
**17**	**1.47**	**1.91**	**2.28**
**18**	**1.02**	**1.47**	**1.57**
19	1.02	1.26	1.35
20	0.75	1.14	1.16
21	0.07	0.93	0.94
22	− 0.61	0.31	0.61
23	− 0.17	0.83	0.83
24	− 0.08	0.87	0.88

Bolded items were either misfit or overfit

### Differential item functioning (DIF)

**Fig. 1:**
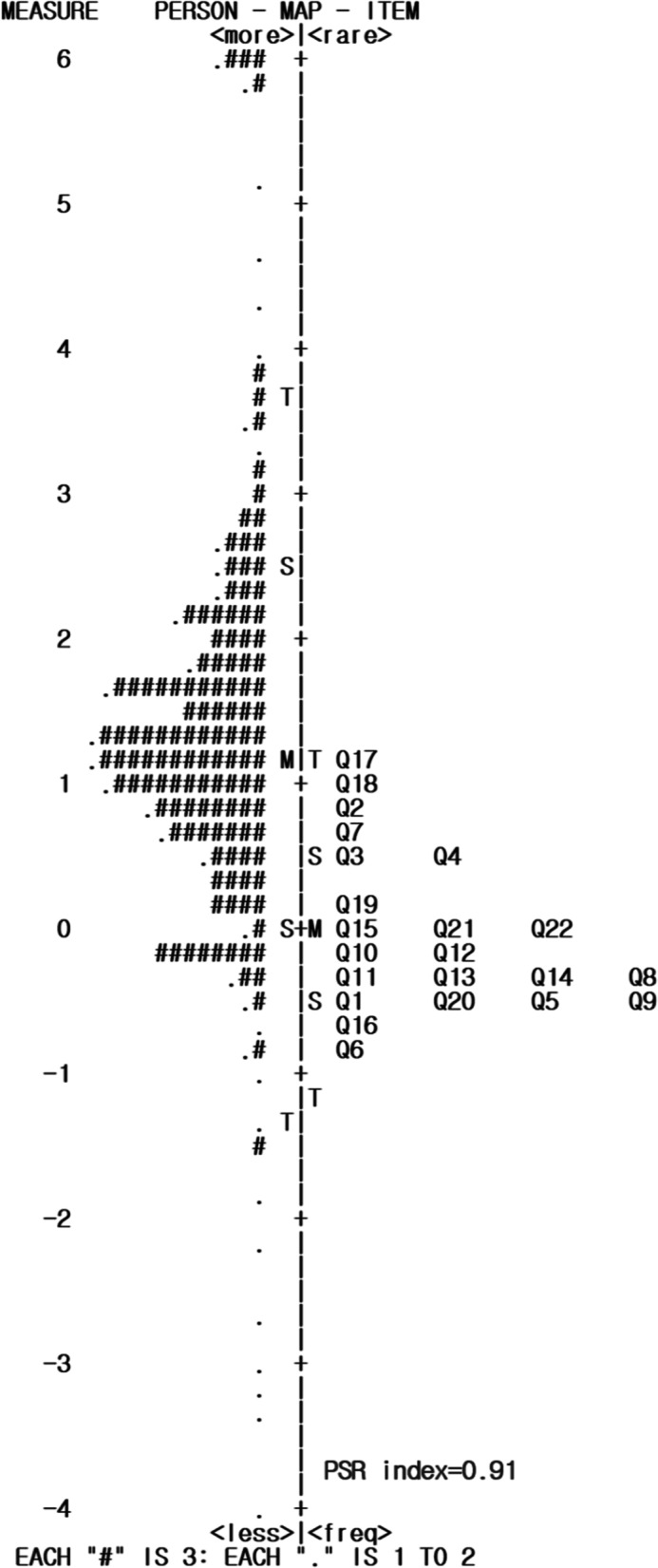
Person-Item map of 22 items of LSS-short form

In order to analyze DIF between female and male respondents, the Rasch rating scale was used to determine if there were any systemically biased LSS-short form items. The remaining 22 LSS items were analyzed to calculate DIF based on sex. As shown in Table 4, item 4 (RW *t* = 1.99; *p* = 0.047) and item 6 (RW *t* = − 2.69; *p* = 0.007) showed statistical difference between female and male participants. However, these two items’ DIF contrast indicated that the absolute values of these items were less than 0.42. In other words, these items were statistically significant, but DIF category status indicated these two items had negligible bias (Class A) [28]. The DIF effect size between female and male participants are shown in Fig. 1. More specifically, the horizontal axis indicated each item, and the vertical axis described the DIF effect size. As shown in Fig. 1, no item’s absolute values were more than 0.42. Furthermore, all 22 items of LSS-short form were suitable for measuring individual leisure satisfaction levels.

**Table 4:** Summary of biased items by DIF

Items No	RW *t*	*p-*value	DIF contrast	DIF category
1	0	1	0	A
2	2.1	0.0361	0.28	A
3	− 0.69	0.4895	− 0.09	A
4	1.99	0.047	0.27	A
5	− 0.58	0.5655	− 0.08	A
6	− 2.69	0.0074	− 0.41	A
7	0.54	0.5912	0.07	A
8	− 1.08	0.2825	− 0.15	A
9	0	1	0	A
10	− 0.53	0.5944	− 0.08	A
11	− 0.62	0.5351	− 0.09	A
12	0	1	0	A
13	− 0.47	0.6365	− 0.07	A
14	− 0.33	0.7448	− 0.05	A
15	1.5	0.1337	0.21	A
16	− 0.7	0.4826	− 0.1	A
19	1.57	0.1164	0.2	A
20	1.38	0.1689	0.18	A
21	− 1.74	0.0825	− 0.24	A
22	− 0.84	0.3989	− 0.12	A
23	0	1	0	A
24	0.17	0.8665	0.02	A

### Person-item map

In an attempt to better understand the reconstructed LSS-short form, the Person-Item map of the Rasch rating scale model was utilized so that both a person’s ability and the item difficulty of participants were integrated to logit value on a single scale. In other words, the Person-Item map indicated the person’s ability of participants and item difficulty map on the same scale. Furthermore, the reconstructed LSS-short form is more suitable measurement to be as close as two distributions of the person’s ability and item difficulty. Figure 1 described that a '•' on the left side means one subject, and a '#' means three subjects. The 'M' on the center line was examined as the mean of a person’s ability and item difficulty on each side. The 'S' was symbolized as one standard deviation from the mean, and 'T' as two standard deviations. As shown in Fig. 1, the left side showed person ability for 436 participants and the right side showed item difficulty for 21 items from the reconstructed LSS-short form. Thus, the top of the left side presented respondents with higher leisure satisfaction levels while the LSS-short form items that had higher item difficulty were displayed from top to bottom sequentially on the right side of Fig. 1. Furthermore, a high level of item difficulty indicated participants were more likely to answer, “strongly disagree.” In addition, the distribution of item difficulty was more like to be functioning properly over the person ability distribution of participants as the Person's Separation Reliability index (PSR index) prove it, which was very high at 0.91 [24].

In order to verify the basic hypothesis of the reconstructed LSS-short form, the unidimensionality of 22 items was analyzed. The results revealed an eigenvalue variance of 46.02%, meaning that the reconstructed LSS-short form satisfactory for the basic assumption of unidimensionality. Furthermore, the new version’s local independence and unidimensionality were examined that this version of the reconstructed LSS-short form would be good to utilize.

## Discussion

The LSS-short form has been utilized to evaluate individuals’ leisure satisfaction level [5, 7, 8]. This study used the Rasch rating scale model to determine whether the LSS-short form was appropriate to use for individuals. Furthermore, this study proposed a new version of the LSS-short form by eliminating unsuitable items or replacing items from the LSS.

Prior to applying the Rasch rating scale model, it was necessary to demonstrate that the LSS-short form’s unidimensionality assumptions verified the 24 items as the one primary factor. The questionnaires or tools to measure individual psychological levels are composed of several factors, but ultimately are united into one primary factor. In this study, even if the LSS-short form was comprised of six subscales, the one primary factor would be needed to test the individual’s leisure satisfaction levels. As indicated in the methods section, PCA was applied to examine the unidimensionality assumption, which resulted in verifying the suitability of applying the Rasch rating scale model to this study [23].

It was determined that a five-point Likert-type LSS-short form was an appropriate category to utilize by following the verification of the unidimensionality assumption. As shown in Table 2, the results of this study suggest a five-point Likert-type LSS-short form is relatively appropriate to measure leisure satisfaction levels. Previous research supports the current finding that five-point Likert-type scales were recommended when respondents were the general public, which is matched to the demographic characteristics of the studies’ participants [30]. Another study’s results showed that five-point Likert-type scales provide better quality of data rather than seven-point or 11 Likert-type scales [31].

As indicated, three LSS-short form items were deemed unsuitable for use by measuring the item fit score from the Rasch rating scale model. In order to better understand participants’ leisure satisfaction levels, items that are too confusing (misfit) or items that are too easy (overfit) should be removed. In this study, item 17, “My leisure activities are physically challenging,” and item 18, “I do leisure activities which develop my physical fitness,” were deemed unsuitable as they were confusing questions on the subscale of “Physiological” benefits. More specifically, individuals with low physiological leisure satisfaction levels tended to respond with a high leisure satisfaction, while participants who had high physiological leisure satisfaction levels responded with lower levels of leisure satisfaction. Therefore, it would be advisable to remove these two items from the LSS-short form to avoid overall biased results in leisure satisfaction levels.

The Rasch rating scale model also evaluated unsuitable items by utilizing Differential Item Functioning (DIF), which is the same approach of the Mentel-Haenszel method. DIF determined biased items by measuring the potential ability level of the two groups through comparing responses [26, 27]. In this study, DIF was utilized with male and female participants, who were evenly divided into two groups. It was necessary to estimate how each question could induce favorable or unfavorable answers based on sex. These biased items should be removed to better understand leisure satisfaction levels among participants.

As shown in Table 4, the results indicated that item 6, “My leisure activities provide opportunities to try new things” and item 17, “My leisure activities are physically challenging,” were statistically significant between female and male participants. More specifically, item 6 was favorable to female participants, which means that this biased item induced female respondents to answer higher leisure satisfaction levels (close to strongly agree) even if both female and male participants were assumed to have the same person ability to response questions. Item 17 was biased to be more favorable to male participants. However, this study was judged to extract biased items of DIF based on the DIF effect size [28]. In other words, even if items were statically significant, items would not be removed if they had no effect on actual bias (negligible bias). Thus, these items might be influenced by the questions’ lexical and grammatical choices [28]. In this study, item 6 was statically significant, but its DIF category was class “A,” which indicated that there was no effect of actual bias between female and male participants to response to this question. Item 17 showed the same case as item 6, but it was already eliminated because of item fit score. Furthermore, there were statistically significant items that produced favorable or unfavorable answers, but it was not necessary to removed them due to DIF effect size [28].

Taken together, the findings from the current study showed that two questions from the physiological subscale were unsuitable to measure leisure satisfaction benefits. These results suggest that the physiological subscale might need to be reconstructed. The original 51 items from LSS provides six items on the physiological subscale. One possible solution might be to replace the two unsuitable items on the physiological subscale with the following questions from the original LSS: “My leisure activities help control my weight” and “My leisure activities help me maintain my energy level”.

## Limitation

It is important to acknowledge the limitations and provide possible future directions. This study was conducted in the south-central U.S. and cannot be generalized to the entire population or to other countries. Future research should consider conducting research from a variety of regions that might yield more powerful and accurate findings. Another important issue was that the lack of sample size might make it hard to generalize the results of the reconstructed LSS-short form. More participants in the future would be helpful to enhance the results. Finally, an important issue was the lack of knowledge about the participants aside from their sex. Future research could provide various demographic characteristics such as age, ethnicity, socioeconomic status, income, education, marital status and so on. It could thus attempt to explain how these demographic characteristics can influence an individual’s choice of leisure satisfaction level.

## Conclusions

As individuals consider leisure time and activities to be more important, it is vital to provide and fulfill their needs so that they continue to enjoy their leisure time. However, leisure and recreation professionals and practitioners might not fully understand why individuals participate in leisure activities or programs. By measuring leisure satisfaction levels through exiting surveys or questionnaires, it is possible to recognize and anticipate participants’ desires through their leisure activities. One widely used survey is the Leisure Satisfaction Scale-short form. The LSS-short form has been utilized to estimate individual leisure satisfaction levels in a variety of disciplines [32, 33]. However, it is necessary to reevaluate whether the LSS-short form can still be useful to measure individual leisure satisfaction levels due to the lack of statistical evidence. In addition, this study found three items were unsuitable to measure individual leisure satisfaction levels. Thus, it was suggested that they be replaced to achieve a better understanding of leisure satisfaction questionnaires that would be validated to another sample to increase external validity. Based on this research, this study anticipates that the reconstructed the LSS-short form should be recommended for evaluating individual leisure satisfaction levels in future studies. Thus, leisure and recreation professionals and practitioners can be equipped to provide and develop effective leisure activities or programs by utilizing new LSS-short form.

## Data Availability

The datasets generated during and/or analyzed during the current study are available from the corresponding authors on request.
